# A systematic review and meta-analysis to compare the efficacy of acyclovir 3% ophthalmic ointment to idoxuridine in curing herpetic keratitis by Day 7 of treatment

**DOI:** 10.1186/s12886-015-0022-2

**Published:** 2015-04-17

**Authors:** Diane E Balderson, Gengqian Cai, Michael A Fries, David M Kleinman, Megan M McLaughlin, Trupti M Trivedi, John I Wurzelmann, Sheila B Young

**Affiliations:** GlaxoSmithKline, Five Moore Drive, Research Triangle Park, Durham, NC 27709 USA; GlaxoSmithKline, 2301 Renaissance Boulevard, King of Prussia, Upper Merion, PA 19406-2772 USA; CSL Behring Biotherapies for Life, 1020 First Avenue, King of Prussia, Upper Merion, PA 19406-0901 USA; Flaum Eye Institute, University of Rochester, Rochester, NY USA

**Keywords:** Acyclovir, Herpetic Keratitis, Geographic ulcers, Ointment, Dendritic Keratitis

## Abstract

**Background:**

This objective of the review and analysis is to demonstrate that acyclovir (ACV) 3% ophthalmic ointment is superior to idoxuridine (IDU) in treating herpetic keratitis (HK) presenting as dendritic and geographic ulcer sub-types.

**Methods:**

Data sources: Publications in human subjects were identified by searching the Ovid MEDLINE database through April 2011, combining medical subject headings (MESH) “Keratitis, Herpetic/” AND “Acyclovir/” limiting by the key words “topical” OR “ointment” and also restricted to MESH “Administration, Topical/” OR “Ointments/”. The results were cross checked with the references used in the Cochrane Database Syst Rev. 1:1–134, 2009 and GlaxoSmithKline clinical documents related to acyclovir.

Study selection: Randomized, double-masked studies in subjects diagnosed with HK with head to head comparator arms of ACV ophthalmic ointment and topical IDU that had actual or calculable healing rates at Day seven.

Data extraction: Data independently extracted from identified articles by two authors of this manuscript.

Data synthesis: Data from seven randomized, controlled trials (RCT) evaluating 432 subjects that met inclusion criteria (214 were treated with ACV and 218 were treated with IDU) and had Day seven healing rates calculable. All sub-classified lesions were identified as either dendritic ulcers (n = 185) or geographic ulcers (n = 35). The Cochran-Mantel-Haenszel (CMH) method in Biometrics 10:417-51, 1954 and JNCI 22:719-48, 1959, controlling for study, was performed as the primary analysis using SAS v9.

Homogeneity was assessed using Breslow-Day-Tarone (BDT) test in IARC 1:1-32, 1980 and Biometrika 72:91-5, 1985. The analysis was performed with outliers removed to assess their impact.

**Results:**

ACV showed statistically significant greater odds of healing HK at Day seven in all subjects (Odds Ratio 3.95, 95% CI2.60, 6.00, p <0.0001), in dendritic ulcers (Odds Ratio 4.22, 95% CI: 2.14, 8.32; p < 0.0001) and geographic ulcers (Odds Ratio 5.31, 95% CI: 1.09, 25.93; p =0.0244).

**Conclusion:**

ACV 3% ophthalmic ointment is a valuable intervention for dendritic and geographic corneal ulcers. ACV and IDU were generally well tolerated in the studies reviewed.

## Background

Herpetic keratitis (HK) is a well described and potentially serious corneal disease. It is estimated that up to ten million people globally have been afflicted with HK, and the incidence of herpetic keratitis due to herpes simplex virus is estimated to be roughly 30 per 100,000 people per year. [1] Cases of herpetic lesions of the cornea are a longstanding source of visual disability and have been reported in the medical literature for well over 100 years [1,2]. The main forms of HK lesions are (a) dendritic and geographic ulceration (often referred to collectively as epithelial HK or superficial HK) and (b) stromal keratitis. Epithelial HK usually presents with thin dendritic branching ulcers, thought due to linear spread of the virus to adjacent cells [3]. While dendritic epithelial keratitis is the more common configuration of epithelial keratitis, a macro-ulceration secondary to epithelial infection is also seen and referred to as geographic epithelial keratitis [1]. Stromal keratitis occurs less frequently than the epithelial manifestation, but it often resolves more slowly [4,5]. Morphologically the corneal stroma becomes inflamed while the epithelial surface remains intact. Herpetic Keratitis can be a self-limited condition and resolve without sequalae; however, untreated, such benign cases occur in a minority of outcomes [6]. Mechanical and nonspecific therapies such as debridement and chemical curettage were in use prior to the 1960’s to improve outcomes. Despite these interventions signs of active HK were observed on the cornea for an average of 21 days, and HK remained one of the most important corneal diseases leading to loss of vision [6].

In 1962 Kaufman et al. [6] showed in an uncontrolled trial effectiveness of topical 5-iodo-2′-deoxyuridine—(later called idoxuridine) in treating HK. Over the ensuing two years multiple authors reported on the efficacy of idoxuridine, including five controlled trials. Success, often defined as the resolution of staining, was reported in 50% to 90% of cases, and the time frame for the efficacy assessment was seven days for three of the five controlled trials [6-10]. Success rates for idoxuridine ophthalmic ointment (IDU) generally fall in to the 75% rate based on review of these early studies. In 1962 IDU became the first antiviral agent approved for use in human disease [1]. Since that time newer agents have been developed. Specifically, acyclovir (ACV) 3% ophthalmic ointment was approved in 1981 in the United Kingdom. With prompt attention visual outcomes in patients with HK can be excellent [11]. Currently, ACV is utilized for the treatment of HK as ZOVIRAX ophthalmic ointment in the EU and many additional countries [12]. While ACV is not approved in the United States, IDU is approved in the United States for the treatment of keratitis caused by the virus of herpes simplex [13]. Both medications are topically applied and have similar adverse reaction profiles. Ocular irritation including burning and stinging are commonly seen immediately following application, and a drug-induced superficial punctate keratopathy including corneal epithelial staining can be observed on examination in a minority of patients using these medicines. Interestingly, however, the efficacy profiles for these two medications are somewhat dissimilar. Multiple independent studies have demonstrated that ACV 3% ophthalmic ointment has healing rates superior to IDU for the treatment of this condition [1,14,15]. A meta-analysis of the efficacy between ACV and IDU should address which drug is the more effective therapy in HK. To compare ACV to IDU in healing HK we reviewed well controlled, randomized trials (RCT) that assessed the efficacy of these antivirals through healing on Day seven of treatment in subjects with HK.

## Methods

Methods of analysis and inclusion criteria were pre-specified and documented in a protocol. These followed the PRISMA guidelines [16].

Study selection criteria for the meta-analysis were randomized, double-masked clinical trials in subjects with HK with head to head comparator arms of ACV ophthalmic ointment and topical IDU that had actual or calculable healing rates at Day seven. No language, publication date, or publication status restrictions were imposed.

No restrictions on subjects were imposed. Where possible, subjects were identified as having either dendritic or geographic ulcers.

The Ovid search engine for the Ovid MEDLINE database through April 2011, combining medical subject headings (MESH) “Keratitis, Herpetic/” AND “Acyclovir/” was used to identify publications that were then further limited by the key words “topical” OR “ointment”. Separately, the initial results generated by combining (MESH) “Keratitis, Herpetic/” AND “Acyclovir/” were also restricted to MESH “Administration, Topical/” OR “Ointments/”. All papers were limited to those concerning human subjects.

All unique papers identified above were cross checked with the references utilized in major reviews including the 2009 Cochrane report [1] and in GlaxoSmithKline clinical development documents related to acyclovir.

The resulting studies were reviewed by the team members against selection criteria, and disagreements were resolved by consensus of the team.

Day seven healing rates were extracted independently by two team members (MAF and GQ) and cross checked. When interpreting the publications/reports the following two terms were used interchangeably: “superficial herpetic keratitis” and “epithelial herpetic keratitis”. Additionally, the meta-analysis did not include subjects that were excluded in the final analysis by the publication. Some publications did not provide exact numbers for Day seven healing or cure. In that case, values were based on tables and figures in the articles.

When a subject did not provide data on Day seven of treatment the following rules were applied:

- If the subject provided data showing the disease status before the completion of Day seven, the same disease status was used as his/her disease status for Day seven of treatment;
- If the subject did not provide any data before Day seven, but provided data showing disease status after that, failure of cure was carried forward for the subject at Day seven;
- If the subject was enrolled but did not provide any data on disease status, the subject was excluded from meta-analysis.

Risk of bias of individual studies was low due to selection of RCTs.

The meta-analysis was performed in a sequential manner to control the overall type I error rate at 5%: data from subjects with HK were analyzed first; the analysis of data from subjects with dendritic and geographic ulcers was further performed at the 2.5% level to maintain the overall type I error rate at 5% level. Superiority was defined as statistically significant higher response rate.

To compare the efficacy of ACV to IDU, the Cochran-Mantel-Haenszel (CMH) method [17,18], controlling for study, was performed as the primary analysis. Point estimates and the corresponding 95% confidence intervals (CIs) of the estimated common odds ratio across studies were provided together with the CMH test statistics and p-value for each ulcer type.

To estimate the efficacy of ACV and of IDU, the non-linear mixed effect (NLME) model (logistic regression model, based on maximum likelihood method) fitting treatment as a fixed effect and study as a random effect, was performed. Point estimates and the corresponding 95% CIs for the log odds of Day seven healing rate for both products were derived. The log odds and the corresponding 95% CIs were then back calculated to the scale of odds and healing rate for each treatment.

Homogeneity was assessed using Breslow-Day-Tarone (BDT) test [19,20] based on the odds ratio (OR). The publications deemed as statistical outliers were removed and the analysis was re-conducted to check the potential impact of the outlier on the result.

To summarize the data available, the determination of Day seven healing rates for ACV and IDU was calculated by combining the number of subjects healed over the selected studies, and dividing by the total number of corresponding subjects treated with that antiviral over the selected studies. The 95% CIs for the healing rates for each treatment group were also calculated using a normal approximation. All analyses were performed with SAS v9.

Safety data from the publications in the meta-analysis was reviewed and summarized. No formal analysis of safety was performed.

## Results and discussion

### Publication review

**Figure 1:**
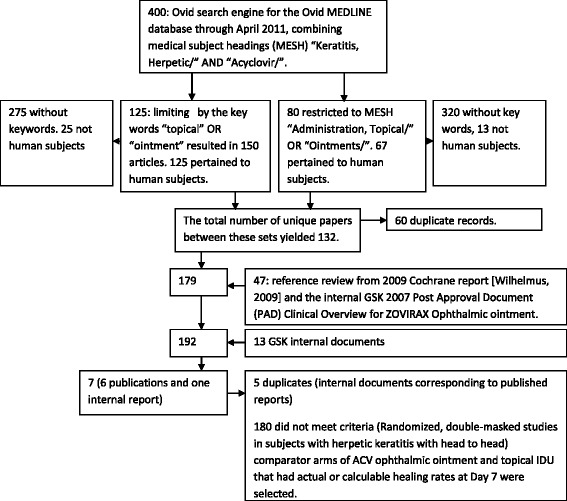
The flow of information through the systematic review.

The flow of information through the systematic review is displayed in Figure 1.

Using the Ovid MEDLINE database through April 2011, combining MESH “Keratitis, Herpetic/” AND “Acyclovir/” resulted in 400 peer reviewed publications. Limiting these by the key words “topical” OR “ointment” resulted in 150 articles. Of these, 125 pertained to human subjects. Similarly, when the 400 articles were restricted to MESH “Administration, Topical/” OR “Ointments/”, 80 articles remained. Limiting these 80 papers to human subjects yielded 67 papers. The total number of unique papers between these sets yielded 132.

Attention was then placed on selected review articles including the 2009 Cochrane report [1] and GSK documents. The reference list from these documents was cross referenced with the above set of 132 papers to identify 47 additional articles discussing the clinical use of ACV ophthalmic ointment that were not captured in the primary literature search. The total number of peer reviewed publications reporting on the clinical use of ACV ophthalmic ointment was 179.

Searching GSK archives located a further 13 clinical reports.

Randomized, double-masked studies in subjects with HK with head to head comparator arms of ACV 3% ophthalmic ointment and topical IDU were then selected from the 179 publications and from the 13 GSK clinical reports. Those that had actual or calculable healing rates at Day seven were used for the primary efficacy analysis. Ultimately, 6 publications and 1 unpublished GSK summary report (Table 1) were identified for the meta-analysis to support primary efficacy. Four of the published study reports had corresponding GSK documentation. When these publications were compared to the GSK reports, safety and efficacy information was consistent and complete between the two different reports of the same clinical trial, and therefore the publication was used as the source for this meta-analysis.

**Table 1:** **Definition of endpoints and cure/healing of the publications/report identified by systematic review**

**Article**	**Definition**
Colin [21]	Endpoints: Number of cures during treatment period, mean time to cure.
	Definition of cure: the absence of epithelial ulceration after instillation of fluorescein using biomicroscopic examination.
Collum [22]	Endpoints: Days to heal and total number of subjects healed.
	Definition of healing: Ulcers were considered to have healed when there was no fluorescein uptake. Ulcers that did not show improvement by day 4 were treated with ara-A ophthalmic ointment and withdrawn.
Coster [23]	Endpoints: Treatment failures, number of days it took ulcers to heal.
	Definition of healing: no epithelial defect demonstrable with Rose Bengal and fluorescein staining
Hamard [24] GSK Report	Endpoints: cumulative cure rate, average healing time.
	Definition of healing: Resolution of the ulcer based on fluorescein staining (inferred).
Kitano [25]	Endpoints: comparing treatment efficacy between study groups.
	Definition of healing: Based on ulcer appearance over time; reported by categories, as follows: Excellent (ulcer disappeared within 7 days), Good (ulcer disappeared within 14 days or reduced by 50% within 7 days), Fair (ulcer reduced by 50% within 14 days), None (ulcers not reduced by 50% within 14 days).
Klauber [26]	Endpoints: Cumulative healing rates.
	Definition of cure: The keratitis was judged to have been cured, at the time when there were no demonstrable epithelial defects by fluorescein and Rose Bengal and with the disappearance of the stromal affection and injection
McCulley [27]	Endpoints: Corneal epithelial healing is the primary measurement of efficacy.
	Definition of healing: the observation of the absence of fluorescent staining and/or faint, segmented staining (“ghost figures”) in the area of previous corneal ulceration within the therapy period without recrudescence.

Publications that were used are Colin [21]; Collum [22]; Coster [23]; Kitano [25]; Klauber [26]; and McCulley [27].

**Figure 2:**
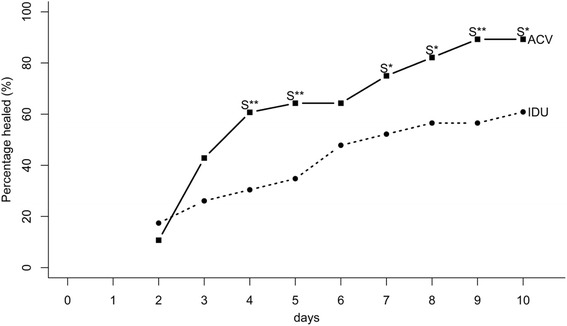
Hamard [24] Day Seven healing rate graph.

The unpublished GSK summary report will be referred to as Hamard [24]. Relevant data is reproduced in Figure 2.

Following the completion of this review, the 2010 update to the Cochrane Review [28] was published. It was analyzed for significant changes or revisions to the information included for acyclovir; none were determined to have an impact on the outcome of the original search or the meta-analysis plan or results, or any conclusion based on information from the 2009 document [1]. Furthermore, none of the selected studies included subjects without epithelial disease, thus healing rates for stromal keratitis were not evaluated in this meta-analysis. The total number of subjects was 432, with 214 receiving ACV and 218 receiving IDU. Regarding dosing, it was generally consistent across the studies with each drug being used five times per day through lesion resolution or 14 days.

### Efficacy data

Seven-day healing rates, defined as proportion of study subjects healed at seven days after study entry, were chosen as the primary outcome measure for this analysis. The choice of Day 7 was influenced by the natural history of the disease, its use as a time point in head to head clinical trials, and the fact that resolution of HK in seven days is a favorable outcome and a reasonable therapeutic goal [1]. In further support of Day 7 as the key endpoint for this meta-analysis untreated HK can take up to three weeks to resolve making resolution within a week a therapeutic success, and many of the original idoxuridine studies utilized a day seven endpoint in their analyses [6,10]. Table 1 is the summary of definition of endpoints and healing from the publications identified by the systematic review.

Table 2 Summary of healing rate for HK, dendritic and geographic ulcers at Day 7 reports the detailed day 7 healing rates for each individual report.

**Table 2:** **Summary of healing rate for HK, dendritic and geographic ulcers at Day 7**

**Study**	**Healed/Total (%)**					
	**Herpetic keratitis**		**Dendritic ulcers**		**Geographic ulcers**	
	**ACV**	**IDU**	**ACV**	**IDU**	**ACV**	**IDU**
Colin [21]	19/25 (76)	11/27 (41)				
Collum [22]	29/30 (97)	6/29 (21)	29/30 (97)	6/29 (21)		
Coster [23]	27/29 (93)	24/30 (80)	27/28 (96)	22/26 (85)	0/1 (0)	2/4 (50)
Hamard [24]	21/28 (75)	12/23 (52)				
Kitano [25]	40/54 (74)	26/55 (47)				
Klauber [26]	12/18 (67)	6/20 (30)	8/10 (80)	5/10 (50)	4/8 (50)	1/10 (10)
McCulley [27]	19/30 (63)	18/34 (53)	16/26 (62)	17/26 (65)	3/4 (75)	1/8 (12)
Summary 95%	167/214 (78)	103/218 (47)	80/94 (85)	50/91 (55)	7/13 (54)	4/22 (18)
CI of summary	(72%, 84%)	(41%, 54%)	(78%, 92%)	(45%, 65%)	(27%, 81%)	(2%, 34%)

In keeping with many clinical trials assessing therapy for HK, the diagnosis in these studies was based on the clinical appearance of the cornea. Although it may appear optimal to only analyze cases in which the presence of herpes simplex was confirmed by culture or alternative laboratory testing, it wasn’t possible to break down the study results based on this parameter. For example, Coster [23], Klauber [26], and Hamard [24] used clinical criteria only. In Colin [21], a viral sample was taken for the majority of cases, but no culture results are reported. Collum [22] showed recovery of herpes simplex virus type I in 19 (33%) of 54 conjunctival swabs while complement fixation antibody to HSV type I was present in 48 (96%) samples of sera. Collum [22] also reported a case by case analysis of recovery of virus and titer of complement fixing antibody. McCulley [27] utilized virus cultures from the inferior cul-de-sac and showed a 19.9% positive culture rate. Kitano reported that virus was isolated in 61.5% of cases. None of these four studies that evaluated subjects for presence of virus separated out their aggregate results based on confirmation of virus. Of note, polymerase chain reaction testing was not available at the time these studies were conducted. It was felt that re-evaluating the Collum study, only, based on isolation of virus from the conjunctiva was not necessary. Furthermore, routine management of HK does not require laboratory confirmation prior to initiating therapy, thus the use of the broader population has clinical relevance [15].

Ulcer size is another variable that can affect responses to antiviral therapy. Five studies provided useful information about baseline ulcer size. Coster [23], Collum [22], Colin [21], McCulley [27], and Hamard [24] all presented a comparison of ulcer sizes between the ACV and IDU groups, and the treatment groups were not different based on analysis of the ulcer size between the groups. Kitano [25] and Klauber [26] did not describe subject ulcer size. It was not possible to reevaluate outcomes based on presenting ulcer size, but based on the similarities between study arms regarding ulcer size in the five studies that mentioned it, such an assessment was not necessary. Although knowing corneal drug concentration in drug development is important and would provide additional information relating to efficacy in these cases, neither corneal nor aqueous humor drug levels were provided in these studies [29]. Likewise, sensitivity testing for drug resistance was not performed.

For some studies, all the healing rates were directly described in the report. In other cases derivation was less straight forward and is explained in detail below:

In Coster [23], the total numbers cured at Day seven had to be extracted from the cumulative time course plots of healing and are reported in Table 3. The numbers of subjects healed at different days are tabulated in the publication. Although 30 subjects were enrolled per arm, *“One patient treated with acyclovir failed to present regularly for follow-up, though he responded favourably in that his geographic ulcer had healed when he returned 10 days after beginning therapy.”* As this subject was not included in the analysis in the publication, he was also excluded from the GSK analysis.

**Table 3:** **Number of subjects healed based on Figures** 2 **and** 3 **of** [23]

**Treatment**	**Group**	**Day 6**	**Day 7**	**Day 8**	**Day 9**	**Day 13**	**Day 14**	**Day 25**	**Total**
Acyclovir	Herpetic keratitis	27	**27**	28				29	29
	Dendritic ulcers	27	**27**	28				28	28
	Geographic ulcers		**0**					**1**	1
Idoxuridine	Herpetic keratitis	21	**24**		26	28	30		30
	Dendritic ulcers	19	22	23		25	26		26
	Geographic ulcers	**2**	**2**			**3**	**4**		4

Note: Bolded values were derived from other cells, unbolded cells were determined directly from Figures 2 and 3 of [23].

In Hamard [24], no corresponding publication of these data was found. Total number of subjects cured at Day seven were extracted based on the cumulative frequency plot in the GSK report. Although the word “dendritic” was used in the abstract, the enrolment states “superficial herpetic keratitis”, and geographic ulceration was not an exclusion criterion. Therefore, the disease type in this study appears to be “epithelial” or “superficial” keratitis generally, and not dendritic ulcer only. No breakdown is provided based on dendritic ulcer or geographic ulcer sub-type in this report, and hence was excluded from the sub-type analysis. See Table 2 for the derived outcome and Figure 2 for the original results in the report.

In KIauber [26], the total number of subjects cured at Day seven was extracted based on the cumulative frequency plots in the publication.

**Figure 3:**
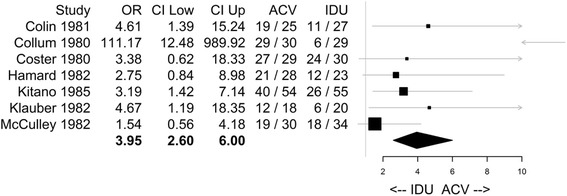
Primary Meta Analysis Result with CMH Method for Herpetic Keratitis**.** CMH: Cochran-Mantel-Haenszel method; OR: Odds Ratio; CI Low: Lower bound of 95% confidence interval; CI Up: Upper bound of 95% confidence interval; ACV: acyclovir; IDU: idoxuridine; Confidence limits were truncated and presented as arrows if they were outside the range 0 to 10; (e.g. the upper confidence interval from Colin [21]). The entire confidence interval for the estimate from Collum [22] was above 10 (with a lower CI of 12.48) and therefore only the lower limit was shown as an arrow. The point estimate of the Odds Ratio for each study is represented by the squares, where the size of the square is proportional to the precision of the estimate. The kite shaped quadrilateral at the bottom of the graph has left and right endpoints at the lower and upper confidence intervals respectively, and the vertices of the kite shape that are its highest and lowest point vertically align at the point estimate of the Odds Ratio from the meta-analysis.

In McCulley [27], the total numbers of subjects by treatment for each ulcer sub-type were available. Based on Figure 3 in the article, at Day seven the total number of HK subjects cured was 19 of 30 for ACV and 18 of 34 for the IDU group.

### Safety data

Brief summaries of reported safety information are provided.

Colin [21] reported that there were four adverse events (AEs) in each group. In the ACV group the AEs were punctate keratitis, punctate keratitis with follicular conjunctivitis, follicular conjunctivitis, and allergy of the eyelids. The IDU group showed two cases of punctate keratitis and two cases of follicular conjunctivitis. Tolerance to the two compounds was similar, and the side effect profile was also similar between the groups.

Collum [22] reported that no serious adverse events (SAEs) were observed, although transient stinging was recorded in eight subjects receiving ACV ophthalmic ointment and in two subjects receiving IDU. Other AE’s in the IDU group were watering of the eyes in two subjects and superficial punctate erosions in six subjects.

Coster [23] reported that there were no AEs which required withdrawal of therapy. Six ACV treated patients and two IDU treated subjects experienced stinging. One subject (treatment not indicated) developed an allergic reaction that subsided on withdrawal of the atropine drops.

Hamard [24] reported that punctate keratopathy was the only adverse effect, seen in four cases receiving ACV ophthalmic ointment and five receiving IDU. There were no hematological or biochemical changes seen in any subject in the study during the course of therapy. Both drugs were well tolerated during this relatively short period of exposure.

Kitano [25] reported that both treatment arms had AEs that were similar in nature, including superficial keratitis (12 in ACV ophthalmic ointment group, seven in the IDU group) and two cases (one in each group) of other external ocular irritation.

Klauber [26] reported that only minor adverse reactions were recorded in both treatments.

McCulley [27] reported that the only significant difference (P < 0.01) in the frequency of development of adverse reactions was found in the incidence of development of superficial punctate epitheliopathy (IDU, 42%; ACV ophthalmic ointment, 11%).

### Results of meta-analysis

The formal meta-analysis results comparing ACV versus IDU are presented Table 4 and in Figure 3. The results of healing rate estimation for ACV and IDU are presented in Table 5.

**Table 4:** **Summary of meta-analysis results on healing of herpetic keratitis at day 7 for acyclovir versus idoxuridine with CMH method**

**Group**	**# of Articles included**	**Total number of Subjects**		**CMH Test for comparing ACV over IDU**		**BDT test for homogeneity of odds ratio**
		**ACV**	**IDU**	**CMH Statistics, P value**	**Common Odds Ratio 95% CI**	**χ** ^**2**^ statistics (d.f.*), p value
Herpetic keratitis	7	214	218	44.54, < 0.0001	3.95 (2.60, 6.00)	17.65 (6), 0.0072
Dendritic ulcers	4	94	91	21.04, < 0.0001	4.22 (2.14, 8.32)	20.71 (3), 0.0001
Geographic ulcers	3	13	22	5.06, 0.0244	5.31 (1.09, 25.93)	4.00 (2), 0.1350
*Sensitivity Analysis (without data from* [22]*)*						
*Herpetic keratitis*	*6*	*184*	*189*	*22.82, < 0.0001*	*2.96 (1.89, 4.66)*	*2.69 (5), 0.7481*
*Dendritic ulcers*	*3*	*64*	*62*	*1.24, 0.2657*	*1.62 (0.69, 3.81)*	*3.10 (2), 0.2127*

*d.f.: degrees of freedom.

**Table 5:** **Results of day 7 healing rate estimation for ACV and IDU with non-linear-mixed effect (NLME) model**

**Group**	**# of Articles included**	**Total number of Subjects**			**point estimate(95% confidence interval)**	
		**ACV**	**IDU**	**Scale**	**ACV**	**IDU**
Herpetic keratitis	7	214	218	Odds	3.70 (2.02, 6.78)	0.89 (0.51, 1.55)
				Log Odds	1.31 (0.70, 1.91)	−0.12 (−0.68, 0.44)
				Healing Rate	0.79 (0.67, 0.87)	0.47 (0.34, 0.61)
Dendritic ulcers	4	94	91	Odds	6.63 (1.43, 30.71)	1.26 (0.32, 4.98)
				Log Odds	1.89 (0.36, 3.42)	0.23 (−1.15, 1.60)
				Healing Rate	0.87 (0.59, 0.97)	0.56 (0.24, 0.83)
Geographic ulcers	3	13	22	Odds	1.17 (0.11, 12.78)	0.22 (0.02, 2.40)
				Log Odds	0.15 (−2.24, 2.55)	−1.50 (−3.88, 0.87)
				Healing Rate	0.54 (0.10, 0.93)	0.18 (0.02, 0.71)
*Sensitivity Analysis (without data from* [22]*)*						
*Herpetic keratitis*	*6*	*184*	*189*	*Odds*	*3.12 (1.55, 6.30)*	*1.06 (0.55, 2.05)*
				*Log Odds*	*1.14 (0.44, 1.84)*	*0.06 (−0.61, 0.72)*
				*Healing Rate*	*0.76 (0.61, 0.86)*	*0.51 (0.35, 0.67)*
*Dendritic ulcers*	*3*	*64*	*62*	*Odds*	*4.08 (0.43, 38.79)*	*2.50 (0.29, 21.88)*
				*Log Odds*	*1.41 (−0.84, 3.66)*	*0.92 (-1.25, 3.09)*
				*Healing Rate*	*0.80 (0.30, 0.97)*	*0.71 (0.22, 0.96)*

Based on the result from the meta-analysis with the CMH method (Table 4), in subjects with HK, the odds ratio (OR) of healing at Day 7 in the ACV treatment group is 3.95 times (95% CI: 2.60, 6.00; p-value: <0.0001) higher than the OR in the IDU treatment group. Since the result based on HK was statistically significant at the 5% level, similar analyses were then sequentially performed for the two ulcer sub-types: dendritic ulcer and geographic ulcer and the results for both sub-types were statistically significant at the 2.5% level (for dendritic ulcer, OR 4.22 (95% CI: 2.14, 8.32; p <0.0001); and for geographic ulcer, OR 5.31 (95% CI: 1.09, 25.93; p = 0.0244). Results from CMH method were also presented in forest plots in Figure 3 Primary Meta Analysis Result with CMH Method for Herpetic Keratitis.

No obvious publication bias was observed based on a funnel plot (not shown).

Homogeneity was evaluated across all studies included in the meta-analysis for HK, as well as across all studies included in the meta-analyses for each of the two ulcer sub-types: dendritic ulcer and geographic ulcer.

The BDT test for the homogeneity of the odds ratios did result in significant p-values for the HK (overall) studies (p = 0.0072), as well as the dendritic ulcer sub-type (p = 0.0001). The test was not significant for the geographic ulcer subtype (p = 0.1357). Collum [22] was deemed a potential outlier based on larger efficacy effect in favor of ACV (Figure 3).

For both the analysis of HK and the dendritic ulcer subtype, a jack-knife analysis was done, where each study was removed in turn and the analysis was re-performed. The results from the BDT test for both the HK group and the dendritic ulcer subtype lost significance when Collum [22] was removed (0.7481 for HK and 0.2127 for dendritic ulcer) while they remained significant when any of the other studies were removed. This finding suggests that Collum [22] was the driver behind the statistically significant BDT test for homogeneity.

The jack-knifed results for the CMH statistic remained significant and consistent with the original analysis in all cases except for one. The result from the jack-knife that lost statistical significance was the analysis on dendritic ulcers with Collum removed (common odds ratio: 1.62; 95% CI: 0.69, 3.81; p = 0.2657), although the direction was consistent and still in favor of acyclovir.

Of note, the HK reanalysis without Collum remained highly significant (common OR: 2.96; 95% CI: 1.89, 4.66; p-value: <0.0001) and was consistent with the analysis with all data for the HK ulcers. This shows the robustness of the conclusion that ACV provided improved 7 day healing rates for HK.

In Collum, the healing rates observed from the ACV (97%, 29/30) and from the IDU (21%, 6/29) arms (and therefore the difference between them) were reasonable given the efficacy outcomes for dendritic ulcers reported in the literature [28,30]. Day 7 healing rates for ACV treated dendritic ulcers reached up to 27/28, or 96% [31]. Day 7 healing rates for IDU treated dendritic ulcers healing rates were as low as 4/20 or 20% [32].

The Collum [22], study was a randomized, double-blind trial, which met the inclusion criteria for the meta-analysis plan and provided day 7 healing rates that were consistent with those in the available literature. Therefore it is not excluded from the primary comparison of interest.

The estimates of treatment effects of ACV and of IDU from the NLME model for different ulcer types are presented in Table 5.

It is also helpful to look at the summary of healing rates for ACV and IDU for HK, dendritic and geographic ulcers at Day 7 using traditional descriptive statistics (Table 2). Summary data showed ACV healed 167/214 cases of HK (78%, 95% CI 72-84%) at Day 7 while IDU healed 103/218 cases (47%, 95% CI 41%-54%) by Day 7. For dendritic ulcers, ACV was effective in 80/94 cases (85%, 95% CI 78-92%) while IDU was successful in 50/91 cases (55%, 95% CI 45-65%). In the setting of geographic ulcers, there was overlap of the 95% CI between the treatment arms, but numerical superiority for AVC (7/13, 54%, 95% CI 27-81%) compared to IDU (4/22, 18%, 95% CI 2-34%). Furthermore, these 7 controlled studies used in the meta-analysis of primary efficacy in herpetic keratitis were also reviewed for inclusion of comparative mean time-to-healing data. Mean time to healing was reported in five of the seven studies (Colin [21], Collum [22], Hamard [24], Kitano [25], McCulley [27]). The mean time to healing for ACV treated eyes ranged from 4.4 days (Collum [22], Hamard [24]) to 7.5 days (Colin [21]) in 167 patients compared with 5.1 days (Hamard [24]) to 9.2 days (Collum [22]) in the IDU treatment groups totaling 168 patients. All 5 papers showed superiority for acyclovir ophthalmic ointment compared to IDU. The mean time to healing was statistically significantly shorter in 3 of these 5 reports (Colin [21], Collum [22], Kitano [25]), and numerically shorter in the other two (Hamard [24] and McCulley [27]). One study (Klauber [26]) reported that mean time to healing was shorter for ACV treated eyes, but the means were not reported directly.

Ten day healing rates for ACV treated eyes was compared to IDU treated eyes as a post hoc analysis. All sources, with the exception of Kitano [25] had sufficient information to derive 10 day rates. Based on the result from the meta-analysis with the CMH method, in subjects with HK, the odds ratio (OR) of healing at Day 10 in the ACV treatment group is 2.99 times (95% CI: 1.68, 5.30; p-value: <0.0001) higher than the OR in the IDU treatment group. Since the result based on HK was statistically significant at the 5% level, similar analyses were then sequentially performed for the two ulcer sub-types: dendritic ulcer and geographic ulcer. The results for the dendritic sub-types was statistically significant at the 2.5% level (for dendritic ulcer, OR 2.40 (95% CI: 1.06, 5.43; p =0.0237); and for geographic ulcer, OR 2.93 (95% CI: 0.71 12.15; p = 0.1129)).

## Discussion

The meta-analysis addressed the objective of comparing efficacy of ACV 3% ophthalmic ointment to IDU in healing HK on Day 7 of treatment. There were seven studies of subjects with HK included in the meta-analysis. From all cases of HK (N = 432) included in the meta-analysis, 185 cases were identified as dendritic ulcers and 35 cases were identified as geographic ulcers. There is not sufficient information to classify the remaining cases as specific ulcer sub-type. ACV 3% ophthalmic ointment had a statistically higher healing rate than IDU in terms of OR (common OR of ACV versus IDU: 3.95; 95% CI: 2.60, 6.00; p < 0.0001). On average the day 7 healing rate was 79% (95%CI: 67%, 87%) for ACV and 47% (95%CI: 34%, 61%) for IDU. Analysis results also demonstrated the statistically significant superiority of ACV over IDU in both ulcer sub-types—dendritic ulcers (common OR of ACV versus IDU: 4.22; 95% CI: 2.14, 8.32; p <0.0001) and geographic ulcers (common OR of ACV versus IDU: 5.31; 95% CI: 1.09, 25.93; p = 0.0244). Regarding dendritic ulcers, on average, the day 7 healing rate was 87% (95% CI: 59%, 97%) for ACV and 56% (95% CI: 24%, 83%) for IDU. For geographic ulcers, the average day 7 healing rate was 54% (95% CI: 10%, 93%) for ACV and 18% (95% CI: 2%, 71%) for IDU. A post hoc analysis of day 10 healing rates showed that the advantage of ACV over IDU remains in HK and the dendritic ulcer subtype remains statistical significant, but the odds ratios are somewhat attenuated. An attenuation of the odds ratio for healing at ten days is not unanticipated as additional time allows for greater assistance from the host’s immune system.

No obvious publication bias was observed based on a funnel plot, however publication bias is still a potential in meta analyses. Outlier analysis did not change the inference of the primary analysis for HK. However, limitations include the modest number of total subjects, and the age of the studies included. Further, only McCulley [27] reported planned evaluation on day 7. For subjects without a day 7 evaluation, healing prior to day 7 was deemed a success, and healing after day 7 was deemed a failure.

The safety profile for ACV ophthalmic ointment was generally favorable. No serious AEs or deaths were reported. The most common AEs reported for ACV and IDU were occasional transient stinging and superficial punctate epitheliopathy. These AE’s were not serious and did not lead to discontinuation of study drug.-ACV and IDU were generally well tolerated in the studies reviewed. IDU is superior over placebo in the management of HK. [6-10] The use of antiviral agents has reduced the frequency of poor visual outcomes in HK [11]. IDU, however, was the first antiviral agent available to physicians, and ACV was approved approximately twenty years later. This meta-analysis shows that ACV has superior outcomes compared to IDU in HK. Antiviral therapy is generally more effective in treating epithelial HK than stromal keratitis, which also requires anti-inflammatory therapy [4,5].

## Conclusions

In conclusion, ACV 3% ophthalmic ointment showed superiority over IDU in the management of HK. Although both medications were well tolerated and have a favorable safety profile, ACV was significantly more efficacious than IDU in HK generally as well as in the subtypes of the disease including dendritic and geographic ulcers. These findings support the efficacy of ACV 3% ophthalmic ointment in HK and further demonstrate the value of this topical therapeutic in HK.
